# Implementation of data-dependent isotopologue fragmentation in ^13^C-based metabolic flux analysis

**DOI:** 10.1007/s00216-017-0339-1

**Published:** 2017-04-07

**Authors:** Teresa Mairinger, Stephan Hann

**Affiliations:** 1 Department of Chemistry, University of Natural Resources and Life Sciences - BOKU Vienna, Muthgasse 18, 1190 Vienna, Austria; 2 Austrian Centre of Industrial Biotechnology, Muthgasse 18, 1190 Vienna, Austria

**Keywords:** ^13^C-based metabolic flux analysis, Isotopologue ratio, Data-dependent fragmentation

## Abstract

A novel analytical approach based on liquid chromatography coupled to quadrupole time of flight mass spectrometry, employing data-dependent triggering for analysis of isotopologue and tandem mass isotopomer fractions of metabolites of the primary carbon metabolism was developed. The implemented QTOFMS method employs automated MS/MS triggering of higher abundant, biologically relevant isotopologues for generating positional information of the respective metabolite. Using this advanced isotopologue selective fragmentation approach enables the generation of significant tandem mass isotopomer data within a short cycle time without compromising sensitivity. Due to a lack of suitable reference material certified for isotopologue ratios, a *Pichia pastoris* cell extract with a defined ^13^C distribution as well as a cell extract from a ^13^C-based metabolic flux experiment were employed for proof of concept. Moreover, a method inter-comparison with an already established GC-CI-(Q)TOFMS approach was conducted. Both methods showed good agreement on isotopologue and tandem mass isotopomer distributions for the two different cell extracts.

**Graphical abstract:**
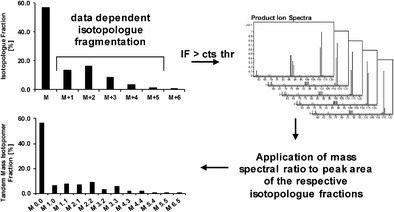
Schematic overview of data-dependent isotopologue fragmentation for acquisition of isotopologue and tandem mass isotopomer fractions

Schematic overview of data-dependent isotopologue fragmentation for acquisition of isotopologue and tandem mass isotopomer fractions

## Introduction

For the characterization of alteration within metabolic networks, the introduction of stable isotope tracers into an organism and the consecutive analysis of the resulting stable isotope-labeling patterns of metabolites has become a key technology to accurately estimate intracellular metabolic rates, i.e., fluxes [1–5]. This isotopic enrichment of intracellular metabolites, typically on the basis of ^13^C labeling, is mostly analyzed on mass spectrometric detectors [6]. This approach, comprising the labeling experiment, the analysis of ^13^C-labeling distributions, and the model-based estimation of in vivo reaction rates, is referred to as ^13^C-based metabolic flux analysis (MFA). With the purpose of increasing the precision on flux estimation calculation, in addition to information on the ^13^C-labeling distribution of the intact molecule, positional information on the stable isotope label is of high interest [7–10] and can be obtained when selectively breaking the carbon backbone using, e.g., tandem mass spectrometry. However, it has to be mentioned that data on isotopologue distribution is sometimes sufficient to draw certain conclusions on activity of certain pathways [11], and hence, tandem mass isotopomer data is not essential for every ^13^C-based metabolic flux experiment, but is rather in need for increasing precision and therefore also resolving very specific metabolic nodes. Different approaches that tackle the challenge of true and precise analysis of the position of stable isotopes in the backbone were published [7, 8, 10, 12–14]. One of the approaches is to generate product ion spectra, also known as parallel reaction monitoring (PRM) mode, where the precursor ion is filtered in the first quadrupole and fragmented in the collision cell, and all generated product ions are monitored in MS2 scans on a high-resolution, accurate mass spectrometer [15].This PRM approach using high mass accuracy mass spectrometry was first employed in the field of quantitative targeted proteomics [15]. We have successfully optimized and implemented this concept to the field of metabolic flux analysis targeting primary metabolites in ^13^C MFA using gas chromatography-quadrupole time-of-flight mass spectrometry [12]. In this GC-approach, an isotopologue selective fragmentation using a high-resolution, high mass accuracy GC-QTOFMS instrument was employed for the first time to obtain information on the position of heavy stable isotope by acquiring the full product ion spectrum. This concept of isotopologue selective fragmentation was also implemented in a recently published study on tracking of the carbon origin of tandem MS CID fragments using liquid chromatography [10]. Another analytical method using this approach, based on liquid chromatography was just recently published by Li et al. based on pure ion intensity data [13].

In the current approach, we are combining parallel reaction monitoring with alternating MS1 (TOF) and MS2 (QTOF) scans employing a data-dependent fragmentation strategy on a high-resolution QTOFMS. A preferred list is scheduled and the fragmentation of isotopologues of metabolites of interest are only triggered, if a certain ion count threshold is reached. This count-dependent fragmentation is considered as a practical filter to reduce data and additionally no time is wasted on transitions, for which the resulting signal intensity would be too low for reliable evaluation due to the limitation of counting statistics. To obtain isotopologue and tandem mass isotopomer distributions within one analytical method, this approach was optimized in terms of cycle time: determination of isotopologue fraction is based on the integration of chromatographic peaks derived from extracted-ion chromatogram (EIC) on the MS1-level, whereas positional information, i.e. tandem mass isotopomer distributions (TMID), can be obtained on the MS2-level by calculating the mass spectral peak ratios of the respective fragment patterns and applying these ratios to the determined isotopologue peak areas. Based on a similar approach a concept using nominal resolution QTRAP hybrid quadrupole ion trap MS employing an MRM transition to trigger the product ion scan was published [14]. However, data-dependent fragmentation of isotopologues *(n −* 1*)* using a high resolution time of flight instrument leads to a significantly lower cycle time, inherent to this mass spectrometer.

As a matter of fact, validation of this novel method based on parallel reaction monitoring combined with data-dependent fragmentation is essential for further application in metabolic engineering and industrial biotechnology. Due to the lack of reference material for cell extracts certified for isotopologue and tandem mass isotopomer ratios, two different approaches for assessment of trueness and precision of the determined isotopologue and tandem mass isotopomer fractions were employed: One approach made use of the concept published by Millard et al. [16]. By cultivating *Pichia pastoris* on 50:50 = ^12^C- to ^13^C-labeled methanol as sole carbon source, the isotopologue distributions of all metabolites are defined by the binomial coefficients found in Pascal’s triangle. This results in a distinct and predictable distribution for each metabolite. In the other approach, we conducted a method inter-comparison with an already published GC-CI-QTOFMS based approach [12] using a cell extract of *P. pastoris*, fed with U^13^C glucose mixed with natural glucose (20:80) [12, 17]. As a proof of concept, the obtained data from both approaches were compared and will be discussed on the basis of citric acid.

## Materials and methods

### Chemicals

The present method targeted the analysis of organic acids, amino acids, and nucleotides, acquired in either positive or negative mode. As a proof of concept, this study focuses only on citrate, which was purchased from Merck (Merck Millipore, Darmstadt, Germany). Methanol and water, both in LC-MS grade were purchased from Sigma-Aldrich (Sigma-Aldrich, St. Louis, MO). Formic acid 98–100% Suprapur was purchased from Merck (Merck Millipore, Darmstadt, Germany). Derivatization reagents necessary for the analytical inter-comparison via GC-CI-QTOFMS can be found in Mairinger et al. [12].

### *P. pastoris* cell extracts

For the *P. pastoris* cell extract with a defined ^13^C distribution, the concept of Millard et al. [16] was employed. This well-defined material was obtained by ISOtopicSolutions (Vienna, Austria) by cultivating the yeast on 50:50 = ^12^C- to ^13^C-labeled methanol as sole carbon source. Methanol employed for this cultivation was checked previously via ^13^C-NMR for exact distribution and the respective binomial coefficients found in Pascal’s triangle were calculated accordingly. The other employed cell extract stemmed from a flux experiment published by Nocon et al. [17]. A *P. pastoris* strain-producing human superoxide dismutase [18], fed with uniformly ^13^C-labeled glucose mixed with natural glucose (20:80), was used for the inter-comparison of the present method and the gas chromatography based approach [12].

### Analysis via LC-QTOFMS, data dependent isotopologue fragmentation

For the analysis of isotopologue and tandem mass isotopomer distributions of primary metabolites a reversed phase column, Atlantis T3 column (150 × 2.1 mm, 3 μm particle size, Waters) with an Atlantis T3 guard column (20 × 4.6 mm, 3-μm particle size, Waters) was used for separation. Mobile phase eluent A consisted of 0.1% (*v*/v) formic acid in water, whereas eluent B was LC-MS-grade methanol. Gradient conditions for RPLC were as follows: 0% B was constant for 2 min, then B was increased to 5% within 6 min and increased stepwise to 10, 20, 40, and 80% B each step within 3 min and finally to 100% B within 4 min and held for 1 min to flush the column, followed by reconstitution of the starting conditions within 0.1 min and re-equilibration with 0% B for 4.9 min, resulting in a total analysis time of 30 min. A flow rate of 0.250 mL min^−1^, an injection volume of 5 μL and a column temperature of 40 °C were applied. Mass spectrometric detection was performed on a 6560 Agilent Ion mobility-QTOFMS system equipped with a dual-spray Agilent ESI Jetstream source. In the acquisition software (Agilent MassHunter Data Acquisition B07.00) the following settings were applied: the acquisition rate was set to 3 Hz in MS1 (i.e., TOF mode) and on MS2 level to 12 Hz, employing the preferred list only option, in the so-called Auto MSMS mode. For other metabolites, depending on the chromatographic peak width, acquisition rate on the MS1 level might need some adjustment, as oversampling of the chromatographic peak is not preferable and would need correction using a smoothing function.

Table 1 shows the scheduled preferred list for the metabolite citric acid (Cit). The reason for omitting the fragmentation of the monoisotopic isotopologue “M + 0” and the fully ^13^C-labeled isotopologue is that the TMID for those two isotopologues is known—for the monoisotopic isotopologue M + 0, there exists also only a monoisotopic fragment; the same conclusion can be drawn for the fully ^13^C-labeled isotopologue. The isolation width of the quadrupole was set to “narrow,” representing a rectangular isolation window of 1.3 m/z centered on the respective mass to charge ratio listed in the preferred list. Collision energy was optimized for the metabolite. As ion count threshold for triggering fragmentation, the value was set to 2000 cts and the maximum number of precursors was limited to six. These settings lead to one TOF spectrum (333 ms) and six MS2 fragmentation mass spectra (83 ms each) within a total cycle time of 0.9 s. Regarding the selection of the triggering threshold, in order to avoid missing data, the value was adapted according to the threshold for ion-counting statistics for the precursor ion, and therefore, a rather low abundant value was chosen. Fragmentation of an isotopologue yielding in a lower ion count will lead to an MS2 spectra consisting mainly of noise, and hence, tandem mass isotopomer fractions that would result from this isotopologue will be set to zero. Additionally, precursors were excluded after three acquired spectra and the list of precursors, that is updated on-line automatically during the run, was released after 0.3 min. The instrument was operated in 2 GHz extended dynamic range mode. It is noteworthy, that triggering fragmentation of the different isotopologues at different time points of the chromatographic peak does not influence the outcome of the respective tandem mass isotopomers. This can be explained by the fact that the isotopologue data is obtained via the chromatographic peak area and isotopologue selective fragmentation at any time point will lead to the same fragmentation pattern since it is concentration-independent.

**Table 1:** Scheduled preferred list of citric acid (cit). The isolation width set to ≈1.3 m/z and Δ retention time to 0.7 min. A mass accuracy of 50 ppm is required for triggering the respective transition

Metabolite	Precursor m/z	Δ m/z [ppm]	Z	RT [min]	Collision energy
Cit M + 1	192.0231	50	−1	4.8	10
Cit M + 2	193.0264	50	−1	4.8	10
Cit M + 3	194.0298	50	−1	4.8	10
Cit M + 4	195.0331	50	−1	4.8	10
Cit M + 5	196.0365	50	−1	4.8	10

**Fig. 1:**
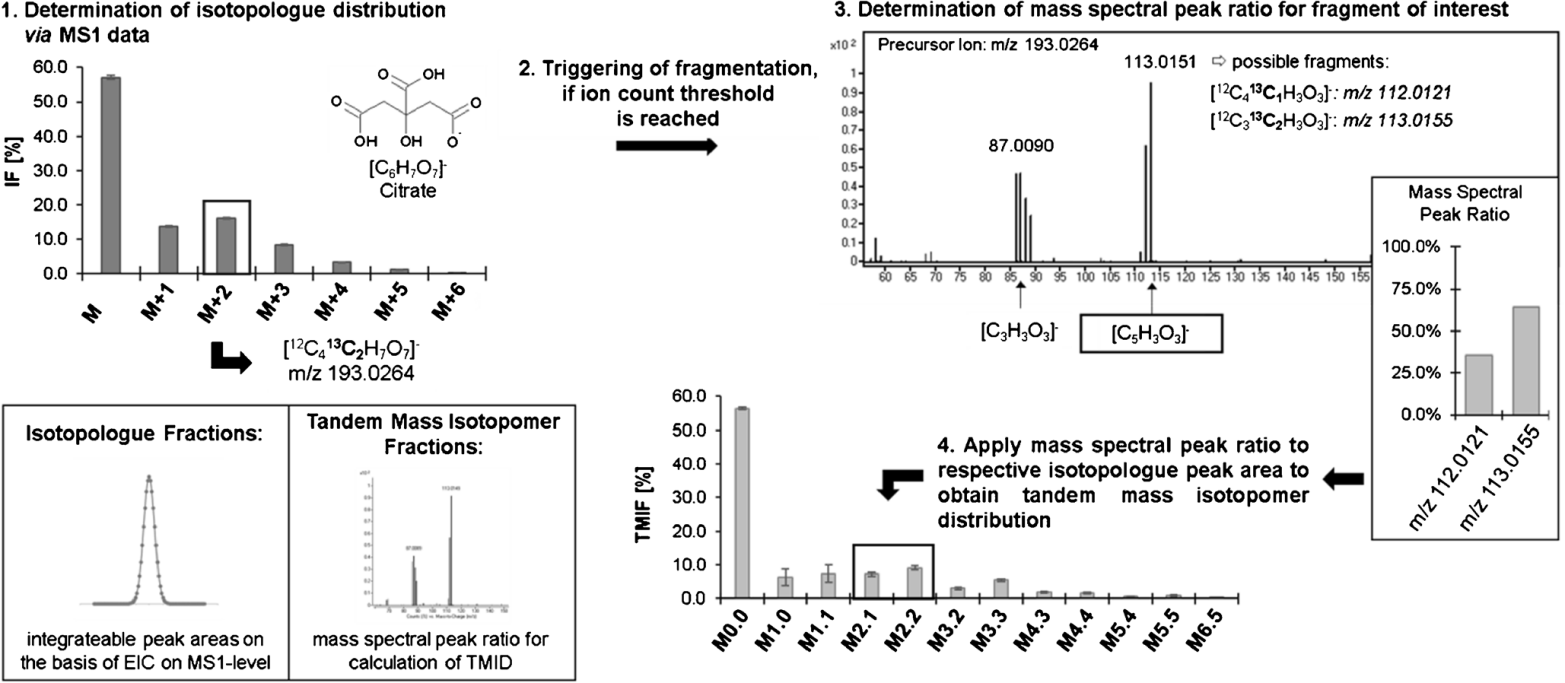
Schematic workflow for the assessment of isotopologue and tandem mass isotopomer fractions of citric acid, employing the method described in the experimental section

For assessment of isotopologue distribution on the basis of MS1 data, data evaluation was performed using Agilent’s MassHunter VistaFlux (B08.00). For evaluation of tandem mass isotopomer fractions, Skyline (3.6.0.10162) was used by setting up a transition list and extracting mass spectra. Additionally, for inspection of certain transitions in more detail, Agilent’s MassHunter Qualitative Analysis (B07.00) was employed. It has to be stated that further improvement of data evaluation of this targeted Auto-MSMS approach is needed to enable high throughput analysis on a routine base. Structural information on product ions were retrieved by in silico fragmentation of the respective compound using MassFrontier 7.0 (Thermo Fisher Scientific Inc.) as well as using information on structural elucidation published recently [10]. Mass spectral peak ratios of selective fragments in MS2 spectra are calculated and multiplied by the respective isotopologue area for determination of the tandem mass isotopomer distribution. A generalized workflow can found in Fig. 1. As for the notation of the tandem mass isotopomer distribution, each fraction is characterized by two digits, where the first is representing the number ^13^C atoms in the precursor ion and the second is indicating the number of ^13^C atoms in the product ion.

## Results and discussion

For the proof of concept, citric acid was used, as for this metabolite GC-QTOFMS and LC-QTOFMS analysis result in the same fragment ion regarding the number of carbons in the backbone. As recently shown comprehensively by Kappelmann et al. [10], the selected fragment ion, containing five carbons in the backbone, is derived from different positions within the precursor ion, which was also shown for fragmentation of citrate in GC-MS by Jung and Oh [19]. It is noteworthy that this fact does not influence the outcome of this analytical inter-comparison.

**Fig. 2:**
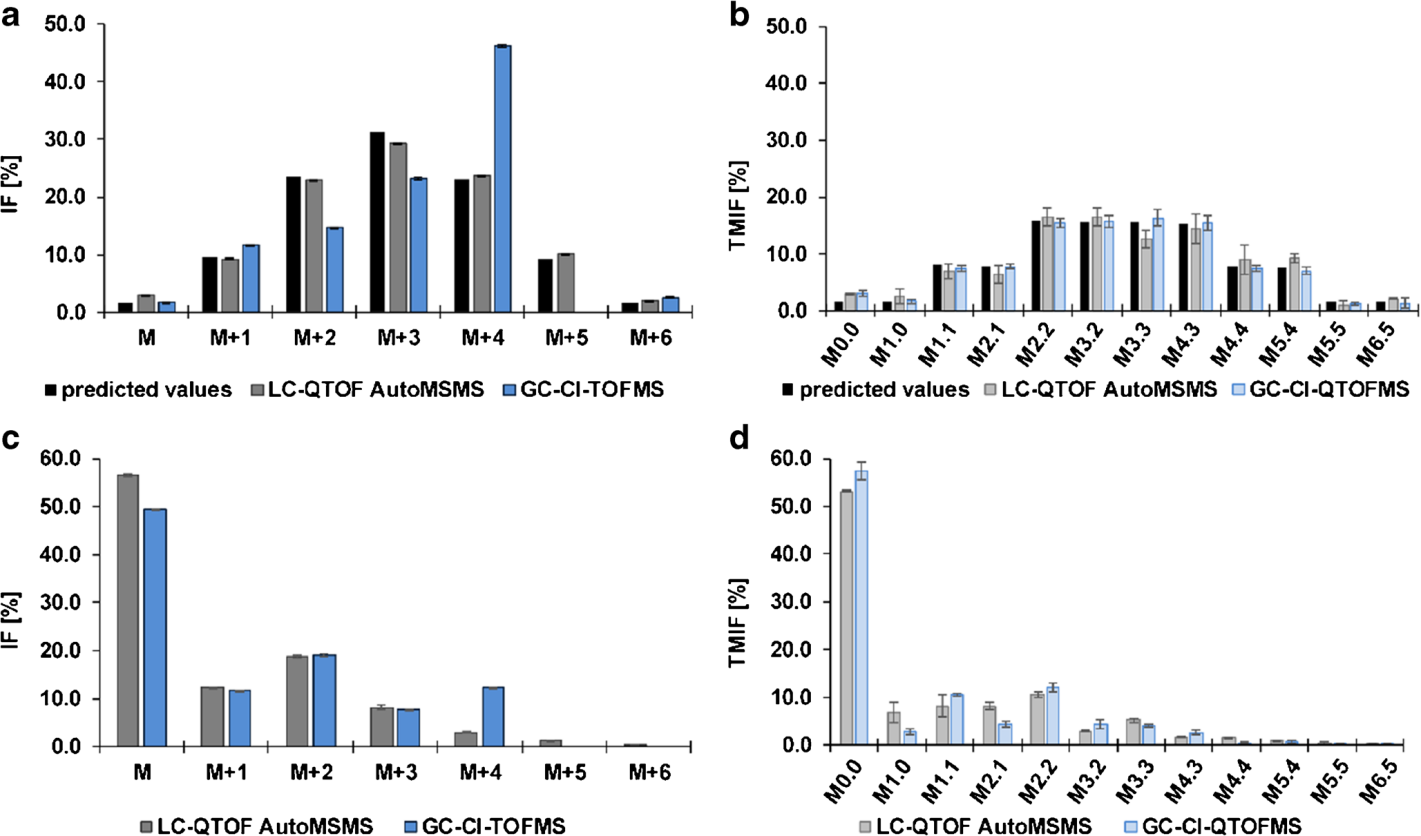
Isotopologue distribution and tandem mass isotopomer distribution of *P. Pastoris* cell extract, fed with either (a,b) 50: 50 = ^12^C_1_ methanol:^13^C_1_ methanol or (c,d) U^13^C glucose mixed with natural glucose (20: 80) [12, 17]. Data obtained from the PRM combined with data-dependent fragmentation LC-MS approach, indicated by “LC-QTOFMS AutoMSMS”, is shown in grey, whereas bars in blue depict the obtained fractions from the already published GC-CI-(Q)TOFMS approach. In Fig. 2a, the predicted values defined by the binomial coefficients found in Pascal’s triangle are depicted in black. As for the TMID in Fig. 2b, TMIFs were calculated according to the predictable distribution following an abstraction of one carbon of a C_6_ backbone. For Fig. 2a, b) *n* = 6, whereas for (c) and (d), three replicates were measured

In order to validate the present method, a *P. Pastoris* cell extract showing a predictable labeling pattern that is defined by the binomial coefficients in the Pascal’s triangle and additionally a *P. Pastoris* cell extract stemming from a ^13^C-based metabolic flux experiment with U^13^C glucose mixed with natural glucose (20:80) as substrate [12], were employed. The results on the methodological inter-comparison of the LC-QTOF AutoMSMS and the GC-CI-(Q)TOFMS approach using these two sample types is shown in Fig. 2. Results acquired in both measurement modes were corrected for interferences of natural isotope distributions using either ICT [20] or the implemented correction of Agilent’s VistaFlux. As can be seen in Fig. 2a, the theoretical/predicted isotopologue distribution (ID), depicted in black, is in good agreement with the measured fractions using the LC-QTOF AutoMSMS approach, shown in dark gray, and also an excellent average repeatability of 0.1% standard deviation and a maximum relative standard deviation of 3.3% for the smallest fraction M + 6. However, employing the GC-CI-TOFMS approach revealed an interference on the isotopologue M + 4 that was not detected before when validating the published method using the natural abundant isotope patterns resulting via the employed derivatization This interference on the isotopologue M + 4 can be also observed in Fig. 2c, where results on the *Pichia pastoris* cell extract from the metabolic flux experiment (20:80 mixture) analyzed by the two different platforms are shown. As can be seen in Fig. 2b, when employing collision induced dissociation in order to obtain positional information of the stable isotope, the interference on the M + 4 isotopologue is not observed anymore, confirming the high selectivity of isotopologue-selective fragmentation via CID in combination with accurate mass spectrometry. In general, the tandem mass isotopomer distribution measured via both approaches are in good agreement with the predicted values, showing also reasonable repeatability also for the lower abundant fractions. For the gas chromatographic approach an absolute bias of the predicted values of tandem mass isotopomer fractions in the range of only 0.04 to 1.44% is observed. However, some fractions obtained by the Auto MSMS approach show a higher bias, e.g., fractions stemming from the isotopologue M + 3 and M + 4, of up to 4.2% for the TMIF of M3.3. This is partly explainable by error propagation, as measurement errors on the respective isotopologue fraction are propagated to the resulting tandem mass isotopomer fractions, since the obtained mass spectral ratio of a fragment on MS2 level is applied to the respective isotopologue fraction.

## Conclusion

A successful proof of concept could be shown for the present approach based on parallel reaction monitoring combined with data dependent fragmentation. The use of a cell extract with a defined non-natural, isotopic labeling pattern as well as a methodological inter-comparison for method validation was proven to be beneficial in order to thoroughly investigate potential interference issues. As could be clearly demonstrated for the GC-CI-TOFMS approach employed for methodological inter-comparison, the use of natural abundant isotope patterns for assessment of trueness of isotopologue and tandem mass isotopomer fractions is not a comprehensively adequate approach, especially concerning naturally low abundant fractions.

The presented method is considered to be highly advantageous due to its time-optimized setup, that is of special interest for metabolites with a higher number of carbons in the backbone (*n* ≥ 10) and, hence, leading to a high number of isotopologues to be fragmented in order to obtain positional information and therefore being challenging in terms of cycle time. Additionally, the new methodology can be also applied to UHPLC-based separations with narrow chromatographic peak widths. The approach is not hampered by any sensitivity issues regarding the acquisition of isotopologue data and has additionally the advantage of retrospective data interpretation on the MS1 level, whereas MS2 is only triggered in case of abundant isotopologue potentially leading to reliable TMIDs.

Next steps will involve further automation of data evaluation on MS2 level and full method validation on a set of cofactors, vitamins, tripeptides, amino acids, and organic acids. Additionally, as this method is applicable to chromatographic peaks with narrower peak widths, it will be translated to an UHPLC method leading to shorter time analysis.
